# Integrated Management of Network Address Translation, Mobility and Security on the Blockchain Control Plane

**DOI:** 10.3390/s20010069

**Published:** 2019-12-21

**Authors:** Younchan Jung, Ronnel Agulto

**Affiliations:** School of Information, Communications and Electronics Engineering, The Catholic University of Korea, 43 Jibong-ro, Bucheon-si, Gyeonggi-do 14662, Korea; ronnelagulto@catholic.ac.kr

**Keywords:** NAT management, smart mobility, security management, blockchain-based management, integrated management

## Abstract

Currently, the dual use of IPv4 and IPv6 is becoming a problem. In particular, Network Address Translation (NAT) is an important issue to be solved because of traversal problems in end-to-end applications for lots of mobile IoT devices connected to different private networks. The vertical model is typically used to solve NAT, mobility and security issues for them. However, the existing vertical model has limitations because it handles NAT, mobility and security management one by one. This paper proposes a Blockchain-based Integrated Network Function Management (BINFM) scheme where the NAT, mobility, and security management are handled at once. The proposed scheme is advantageous in that by using blockchain and the Query/Reply mechanism, each peer can easily obtain the necessary parameters required to handle the NAT, mobility, and security management in a batch. In addition, this paper explains how our proposed scheme guarantees secure end-to-end data transfers with the use of one time session key. Finally, it is proved that the proposed scheme improves performance on latency from the viewpoints of mobility and security compared to the existing vertical model.

## 1. Introduction

One of the main reasons to extend the IP address space in IPv6 is to give Internet of Things (IoT) devices a platform to operate on for solving scalability issues [1,2,3]. The purported 400% increase in growth in the last five years sheds some light on how much exponential IoT growth we can expect to see in the next several decades [4]. However, its slow replacement of IPv4 can cause a problem especially for the demand to construct smart cities in a decade [5]. Recent smart cities need to allow IPv4 network-connected IoT devices to connect each other. As of now, Network Address Translation (NAT) solves connectivity issues for the IPv4 network-connected IoT devices [6,7,8]. NAT has one accessible public address which will be shared among End Nodes (ENs) inside the private network. NAT essentially extends internal addressing from the global IP addressing used over the Internet. NAT provides network resources to get over a shortage of the address space by mapping relatively public IP addresses to private IP addresses. However, the non-standardized characteristics of NAT cause traversal problems especially with the development of peer-to-peer applications for small IoT devices [9].

Considering that IPv6 allows IPv6 network-connected IoT devices to be uniquely addressable, despite the use of NAT and IPv4 private IP addresses, the mobile IoT devices, which enter private networks, must be allowed to solve a connectivity issue. Then, this enables increasing mobility for mobile IoT devices connected to private networks in the smart city on the condition that a smart mobility management is provided [10,11,12].

In recent decades, preserving privacy and ensuring the security of data have emerged as important issues as confidential information or private data may be revealed by powerful data mining tools [13,14,15]. Therefore, if hackers attack a smart city with lots of IoT devices, the outcome could be far more catastrophic. It is argued that IPv6 offers better security solutions than IPv4, largely due to IPSec, with which IPv6 operates. It is known that widespread adoption of IPv6 will make man-in-the-middle attacks significantly more difficult [16]. IPSec, which works on a layer 3 plane, i.e., the network layer, aims to provide application-layer security in batch by means of securing IP-layer. However, it poses a difficult problem to run end-to-end encryption because of its difficult key exchange protocol between end-to-end IoT peers [17]. Small IoT end points will face a burden when they handles security association data to secure the layer 3 datagram services. So this paper is based on the idea that IPSec is difficult to be realized for mobile IoT devices with the private IP addresses.

**Figure 1:**
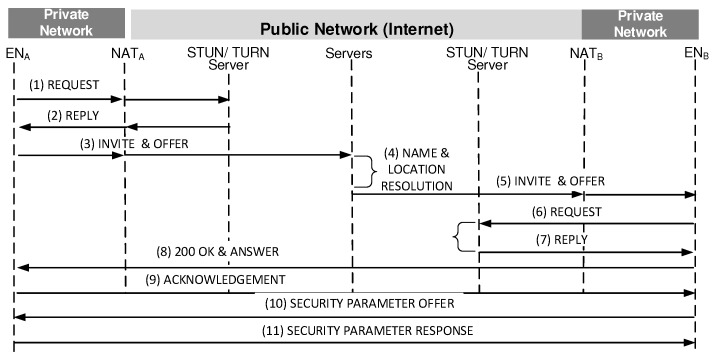
Vertical model for NAT, Mobility and Security management.

As depicted in Figure 1, the existing vertical model starts with the NAT management followed by mobility management. Once the NAT and mobility management are made, the security management procedure begins.

Static assignment of IP addresses gives adversaries significant advantage to remotely scan networks and identify their targets accurately and quickly. As traditional approaches against this attack, the IP address assignment scheme based on DHCP or NAT has been used. However, they are insufficient to provide proactive countermeasures because the IP mutation is infrequent and traceable. Recently, OpenFlow Random Host Mutation (OFRHM), in which the OpenFlow controller frequently assigns each host a random virtual IP that is translated to/from the real IP of the host, has been proposed [18]. The real IP remains untouched, so IP mutation is completely transparent to end-hosts. Implementation of this technique requires two major components: (i) subnet gateways to perform [real IP]/[virtual IP] translation, and (ii) a central management authority that coordinates mutation across the network. Software-defined networking (SDN) provides a flexible infrastructure for developing and managing random host mutation efficiently and with minimal operational overhead. Mobile Edge Computing (MEC) has emerged as a vital solution to offer computing resources at the edge of the network and in close vicinity to the mobile end-users [19]. The concept of MEC was motivated by the unprecedented growth of mobile traffic. Also, as another 5G enabling technology, SDN is complementing the MEC advancement. The MEC environment can substantially benefit from SDN technology. The routing of the ENs’ offloading traffic can be performed in the control plane, which is implemented within the SDN controller. The commonality of OFRHM and MEC is to use SDN as a means and the difference of them is related to the different goals of ENs’ frequent IP mutations and offloading traffic. Differently from SDN-based approaches, blockchain-based decentralized management schemes have been proposed [20,21]. In this paper, we also use the blockchain technology instead of SDN. Also, the goal of this paper is to make the integrated management of NAT, mobility, and security perform efficiently.

In this paper, three issues are addressed for small IoT devices which enter the private networks. First, a smart NAT management is needed in order to manage the private addressing of the local ENs in the private region and solve the NAT traversal issues. Second, the issue of mobility management, which focuses on ENs that use private IP addresses, should be solved. Most of the existing mobility management schemes only deal with the tracking of the location of the EN but not its transport addresses, i.e., the internal private address and mapped NAT address. Therefore, NAT management needs to collaborate with mobility management. Third, this paper solves security issue without the use of IPSec. During a session between two peers, they can use one time session key, which is delivered by the key exchange procedure with the blockchain’s help. Differently from the vertical model, this paper aims to use a Blockchain-based Integrated Network Function Management (BINFM) system where the blockchain control plane functions as a platform to deal with the NAT, mobility and security management in batch. In the BINFM system, the session initiator can obtain the transport address-related and security-related information of the session responder just at the time when the initiator calls the responder. Jung et al. proposed a blockchain-based security management scheme to make a real-time packet key exchange perform better [22]. As a similar approach, for a certain end node, its security-related information are already stored and updated in the blockchain. Therefore, any end node, which wants to establish a new session to its peer, can obtain security parameters to derive the one time session key with the help of the blockchain while the vertical model requires the extra hand shaking procedure of key agreement in order to complete the security management.

The BINFM scheme uses one of the most innovative features of the blockchain, in which there is no central server running. It operates through the network of blockchain control plane that the super nodes (SNs) constitute. Here, the BINFM scheme includes Query/Reply mechanism from the viewpoints of ENs. Using the Query/Reply mechanism, the EN obtains the transport address-related information, which solve the NAT and mobility management, and security-related information from its nearest SN. Therefore, this idea of Integrated Management (IM) gives significantly advantageous effects on NAT, mobility and security controls by reducing the system complexity and latency taken for mobility and security controls. Furthermore, the use of one time session key makes data flow over BINFM system more secure.

The rest of this paper is organized as follows. Section 2 proposes the blockchain-based architecture for smart NAT management. In Section 3, this paper explains how to process a transaction to create a block and query/reply mechanism needed to access the transaction information from the blockchain. Section 4 describes the improvement effects of the proposed management system. This paper concludes in Section 5.

## 2. Blockchain-Based Network Architecture for the Integrated Management

### 2.1. Proposed Network Architecture for Blockchain Control Plane

**Figure 2:**
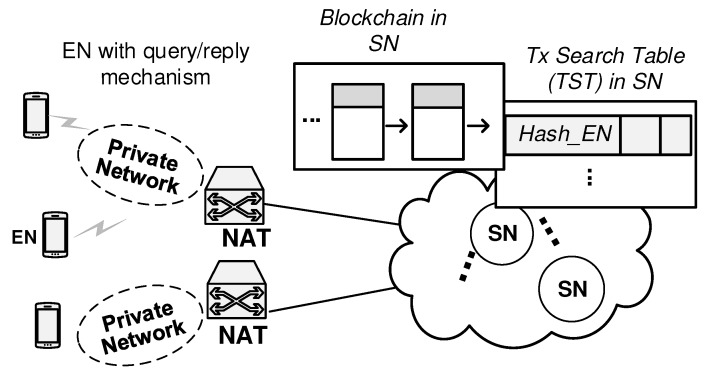
Proposed network architecture for Blockchain-based integrated management.

The blockchain keeps the database associating with the current transport address-related information for every mobile ENs from the NAT and mobility management viewpoints. For a specific application in the $EN_{A}$ behind the private network, a set of transport address-related information is assigned, that is, [private IP address, private source port number, NATed public IP address, NATed port number] = [$IP\_EN_{A}\_Pri$, $Port\_EN_{A}\_Pri$, $IP\_EN_{A}\_NAT_{ED}$, $Port\_EN_{A}\_NAT_{ED}$]. For the corresponding $EN_{B}$ behind the different private network, the transport address-related information of [$IP\_EN_{B}\_Pri$, $Port\_EN_{B}\_Pri$, $IP\_EN_{B}\_NAT_{ED}$, $Port\_EN_{B}\_NAT_{ED}$] are assigned. The role of the blockchain network is to enable both of session initiator and session responder to understand the transport address-related information for each other via the blockchain network. Query(to Blockchain)/Reply (from Blockchain) mechanism enables the EN to obtain information necessary for integrated management. In order to give an answer for this Query, the blockchain network requires to support the registration process that the EN’s latest state information enter the blockchain. Figure 2 shows the BINFM network architecture, which can be explained as follows:

1. The EN is identified by the hash address derived from the public part of a public-private cryptographic key. The private part of the key is under the control of the EN.
2. The EN is responsible to update its Integrated Management (IM)-related information by pushing it into the blockchain. When an EN changes its private network, it sends a new registration transaction, that is, ToALL Tx, to the nearest super node (SN). After an SN receive the transaction message, it broadcasts the message to all SNs. Each transaction message contains several data fields for NAT, mobility and security management, which will be described in the next section.
3. The SN collects new ToALL Txs into a block and performs on solving the proof-of-work for its block. When an SN finds a proof-of-work for the ToALL Tx, it broadcasts the resultant block to all SNs. SNs imply their acceptance of the block by working on creating the next necessary block in the chain, using the hash of the accepted block as the previous hash. SNs will always keep working on extending it. The latency to extend a new block in the blockchain is closely related to the latency that takes the registration process to be completed. Therefore, this latency needs to be reduced as much as possible. Therefore, in this paper, it is assumed that next necessary block is created every 1 second on average and extended to the existing blockchain. This assumption is based on our experimental results using our blockchain testbed.
4. The EN uses the Query/Reply mechanism to obtain the peer’s IM-related information from the blockchain. When an EN initiates to setup a session to its peer EN, it needs the peer EN’s ToALL Tx information. To obtain this Tx information, it sends a query message to the nearest SN to gather the peer EN’s transport address to reach there. Then, the SN searches the Tx Search Table (TST) and finds the corresponding transaction data from its blockchain. Then, it returns the requested transaction information to the EN.

### 2.2. Requirements for the Proposed System

**Figure 3:**
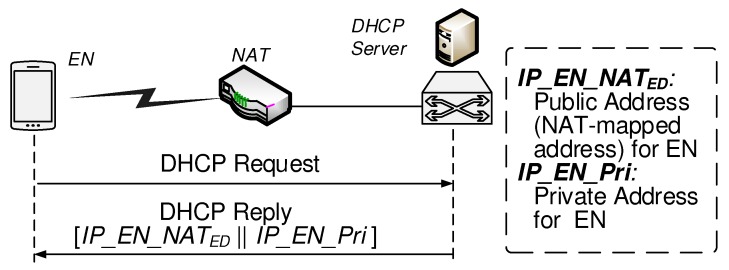
Obtaining public NAT address during private address assignment stage.

Private IP addresses must be configured automatically for new ENs that move from one network to another. Dynamic Host Configuration Protocol (DHCP) server maintains a pool of private IP addresses and leases an address to any DHCP-enabled EN when it starts up on the network. A DHCP-enabled EN, upon accepting a lease offer, receives a valid private IP address for the private network to which it is currently connecting. There are additional parameters that a DHCP server is configured to assign to ENs. In the proposed BINFM scheme, the NAT address ($IP\_EN\_{NAT}_{ED}$, which is one accessible public address that will be shared among ENs inside the private network, is included in those parameters DHCP server offers. Figure 3 shows that the DHCP reply message contains the offered private address and the NAT address which will be used as the EN’s source address when its packet enters the public network.

**Figure 4:**
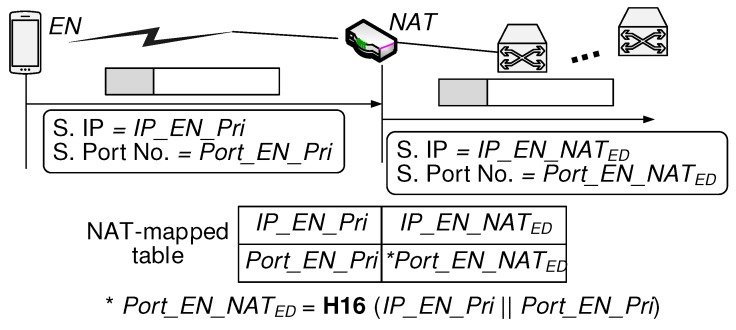
Public NAT port number determined as a function of EN’s private IP address and port number.

When EN sends a packet using its source private IP address and port number (IP_EN_Pri: Port_EN_Pri) to destination IP address and port number (IP_Dest: Port_Dest), the NAT creates a map for EN’s private address and port number (IP_EN_Pri: Port_EN_Pri) by assigning public $IP\_EN\_NAT_{ED}$ and $Port\_EN\_NAT_{ED}$ as public address and port number, respectively. Therefore, incoming packets from [IP_Dest: Port_Dest] destined to [$IP\_EN\_NAT_{ED}$: $Port\_EN\_NAT_{ED}$] are forwarded to [IP_EN_Pri: Port_EN_Pri]. As depicted in Figure 4, the BINFM scheme requires the important condition that $Port\_EN\_NAT_{ED}$ should be derived from the hash function of IP_EN_Pri and Port_EN_Pri. EN is aware that NAT devices use the NAT port assignment function of **H16** where the first 16 bits are taken from the hash value.

## 3. Blockchain-Based NAT, Mobility and Security Management

### 3.1. Smart Wallets for the End Nodes

**Figure 5:**
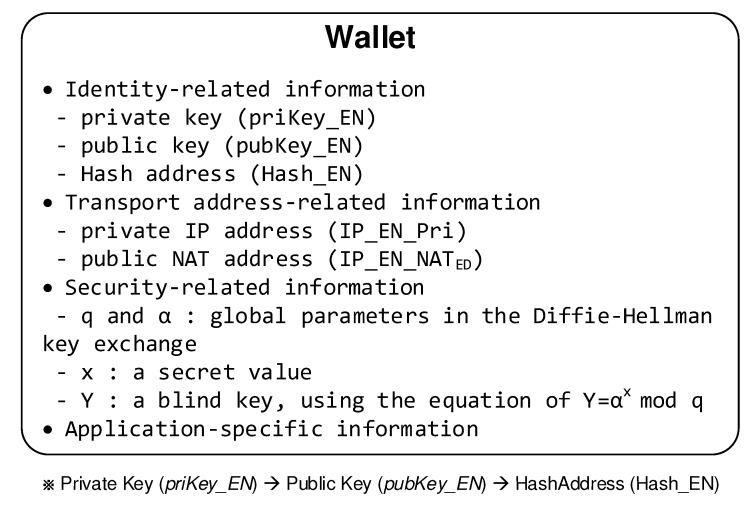
Smart wallet information.

ENs such as small IoT devices have smart wallets. As shown in Figure 5, smart wallet contains its own identity-related, transport address-related and security-related information. So the EN is responsible for registering this information with the blockchain maintained in the SNs. To obtain this information for the other side from the blockchain, the EN uses Query/Reply mechanism. Therefore, each side can easily obtain the necessary parameters of the other side required to handle the NAT, mobility and security management to establish and maintain a secure session between two peers which entered two different private networks. Here, ‘smart’ wallet means that the EN takes advantage of the blockchain’s merits without maintaining the blockchain data structure.

### 3.2. BINFM Transactions and Registration Procedure

**Figure 6:**
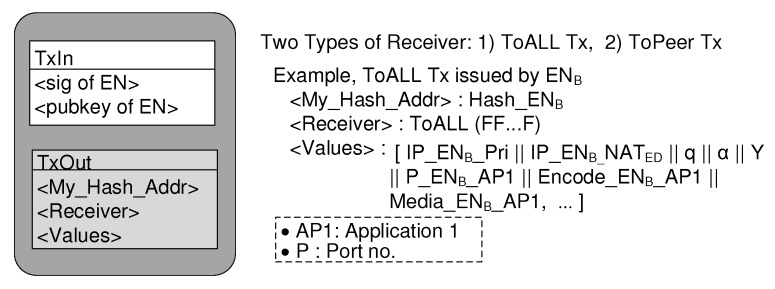
BINFM transaction structure.

Each EN’s latest state information resides in its own wallet. however, all ENs’ information are stored in a distributed database called the blockchain, which stores a secure list of all ToALL transactions sent by them. The BINFM transaction is defined as the EN’s state record during the period of temporally assigned private IP address. Therefore, the transaction change rate is the same as the private IP address change rate. This means that EN sends its ToALL Tx to the network whenever it moves and obtains a new private IP address. When a handover occurs during a call session between two ENs, the EN, which changes its private IP address, also issues new ToALL Tx to the network. The transaction consists of Transaction Input (TxIn) and Transaction Output (TxOut). The TxIn contains the signature and the public key computed from the EN’s private key which creates the transaction. The first field of TxOut contains the hash address that identifies the owner of this transaction. Figure 6 explains the ToALL Tx structure.

**Figure 7:**
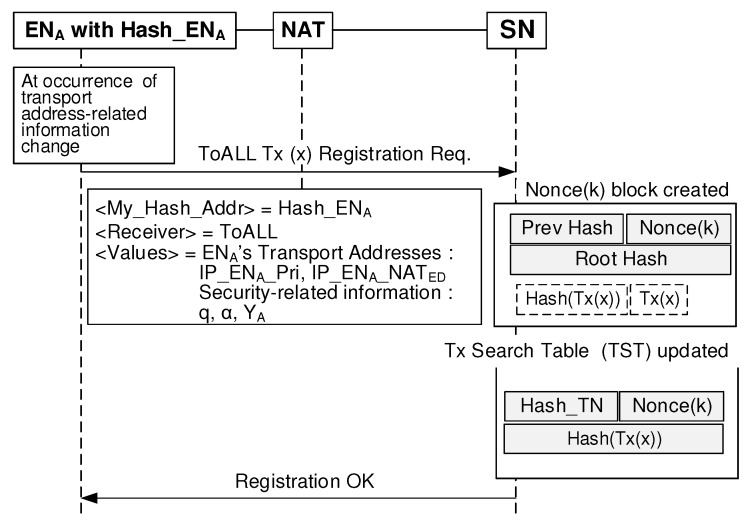
BINFM registration process.

Figure 7 shows the registration procedure of the IM information. Each EN updates their state information including the transport address information by sending ToALL Tx whenever it moves to the new private network. The first SN in the network that receives the Tx verifies the sent Tx if it is a valid Tx. If the Tx is correct, the SN relays it to other SNs in the network.

### 3.3. Blockchain-Based Integrated Management Procedure

**Figure 8:**
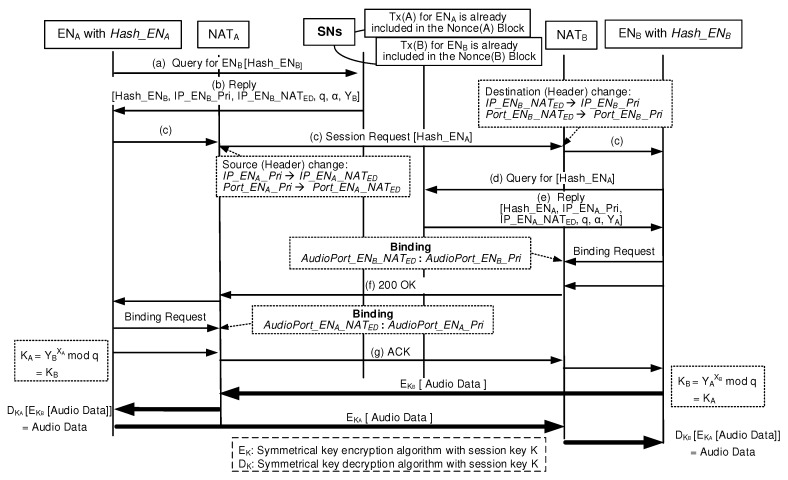
Blockchain-based NAT, mobility and security management procedure.

Figure 8 shows the proposed BINFM-based Integrated Management procedure to establish a secure session between two peers that stay in two different private networks. When $EN_{A}$ with the $Hash\_EN_{A}$ wants to establish a session with $EN_{B}$ with the $Hash\_EN_{B}$ (session responder), $EN_{A}$ first uses the Query/Reply mechanism. $EN_{A}$ sends (a) Query message which contains $Hash\_EN_{B}$ to the nearest SN. When an SN, which has the blockchain information, receives the Query, it seeks the corresponding Tx for $Hash\_EN_{B}$ with the help of TST. The SN sends back (b) Reply message containing the Tx information of $EN_{B}$, that is, global parameters of *q* and α and $EN_{B}$’s blind key ($Y_{B}$) as well as the transport address-related information ($IP\_EN_{B}\_NAT_{ED}$ and $IP\_EN_{B}\_Pri$). Here, $Y_{B}=\alpha^{X_{B}}mod\;q$ where $X_{B}$ is a secret value of $EN_{B}$. Now, $EN_{A}$ can send (c) Session Request message to $IP\_EN_{B}\_Pri$ via $IP\_EN_{B}\_NAT_{ED}$. This message contains $EN_{A}$’s hash address of $Hash\_EN_{A}$. When $NAT_{B}$ receives the packet, it translates the destination IP address and destination port number of the datagram $NAT_{A}$ sent, as $IP\_EN_{B}\_Pri$ and $Port\_EN_{B}\_Pri$. When $EN_{B}$ receives the Session Request message, it extracts the $EN_{A}$’s hash address of $Hash\_EN_{A}$ from the message. Now, the $EN_{B}$ sends (d) Query message which contains the $Hash\_EN_{A}$ to the nearest SN. When an SN receives the Query, it seeks the corresponding ToALL Tx published from the $Hash\_EN_{A}$. The SN sends back (e) Reply message containing the Tx information for $EN_{A}$. Then, $EN_{A}$ obtains global parameters of *q* and α and $EN_{A}$’s blind key ($Y_{A}$) as well as the physical addresses of $IP\_EN_{A}\_NAT_{ED}$ and $IP\_EN_{A}\_Pri$. Here, $Y_{A}=\alpha^{X_{A}}mod\;q$ where $X_{A}$ is a secret value of $EN_{A}$. Now, $EN_{B}$ is ready to send its datagrams to $IP\_EN_{A}\_Pri$ via $IP\_EN_{A}\_NAT_{ED}$. When $NAT_{A}$ receives those datagrams, it translates the destination IP address and destination port number as $IP\_EN_{A}\_Pri$ and $Port\_EN_{A}\_Pri$.

From security management viewpoints, $EN_{A}$ and $EN_{B}$ maintain $X_{A}$ and $X_{B}$, respectively. After each Query/Reply procedure, $EN_{A}$ and $EN_{B}$ are ready to use $Y_{B}$ and $Y_{A}$, respectively. Then, $EN_{A}$ computes the one time session key of $K_{A}$ using the equation of ${Y_{B}}^{X_{A}}\;mod\;q$ while $EN_{B}$ computes the one time session key of $K_{B}$ using the equation of ${Y_{A}}^{X_{B}}\;mod\;q$. Here, $K_{A}$ is equal to $K_{B}$. Now, $EN_{A}$ can encrypt its datagrams using the session key which results in $E_{K_{A}}$[Audio Data] where $E_{K_{A}}$ is any symmetrical key encryption algorithm with the key $K_{A}$. Therefore, $EN_{A}$ sends the encrypted datagrams to $EN_{B}$. When $EN_{B}$ receives the encrypted datagrams from $EN_{A}$, it can decrypt those datagrams using the session key $K_{B}$ which results in $D_{K_{B}}$[$E_{K_{A}}$[Audio Data]] = [Audio Data] where $D_{K_{B}}$ is any symmetrical key decryption algorithm with the key $K_{B}$. As a result, bidirectional session traffic travel over the established secure sessions. Therefore, our blockchain-based scheme easily solves the problem of handling complex issues of NAT, mobility and security management. This advantage results from the fact that each peer can obtain the necessary parameters for peer-to-peer session establishment via a simple Query/Reply mechanism between an EN and its nearest SN.

### 3.4. Blockchain-Based Mid-Call Mobility Management Procedure

**Figure 9:**
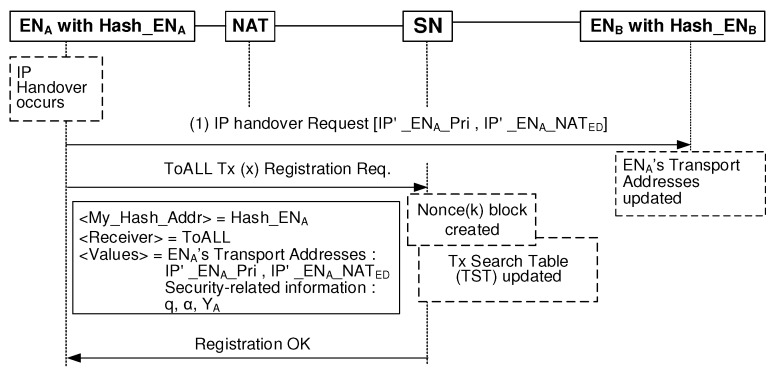
Blockchain-based mid-call mobility management procedure.

The mid-call mobility management is needed when either $EN_{A}$ or $EN_{B}$ changes its local private network during an on-going session. Figure 9 shows the mid-call mobility management operation for the case that $EN_{A}$ changes its network during the on-going session with $EN_{B}$. When $EN_{A}$ confronts with IP handover, it first sends IP handover Request message to the $EN_{B}$. This massage contains a new transport address-related information, that is, [$IP^{\prime }\_EN_{A}\_NAT_{ED}$ and $IP^{\prime }\_EN_{A}\_Pri$]. Next, a new ToALL Tx registration procedure starts to update $EN_{A}$’s state information on the blockchain. When $EN_{B}$ receives the Request message, it immediately uses the updated transport address information for $EN_{A}$. Then, both can keep on going the existing bidirectional session.

### 3.5. Key Renewal Process during a Session

**Figure 10:**
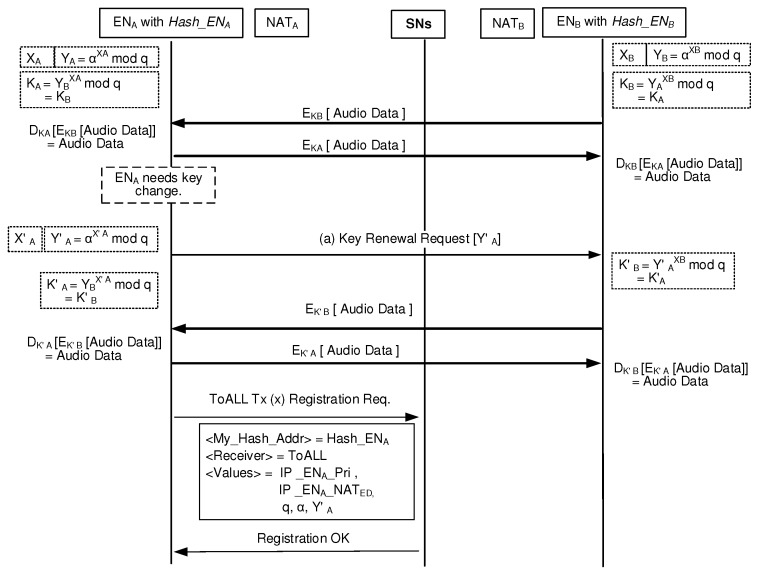
Key change event during a session.

From security management viewpoints, any side can initiate to change the one time session key even during an on-going session. Figure 10 shows the key change operation for the case that $EN_{A}$ needs to change its one time session key during the on-going session with $EN_{B}$. As a key change initiator, $EN_{A}$ generates new secret value ${X^{\prime }}_{A}$ and computes ${Y^{\prime }}_{A}=\alpha^{{X^{\prime }}_{A}}mod\;q$. Also, $EN_{A}$ prepares new one time session key ${K^{\prime }}_{A}={Y_{B}}^{{X^{\prime }}_{A}}mod\;q$. Then, $EN_{A}$ sends the Key Renewal Request message, which contains the blind key ${Y^{\prime }}_{A}$, to $EN_{B}$. Once $EN_{B}$ receives ${Y^{\prime }}_{A}$, it computes the one time session key of ${K^{\prime }}_{B}$ using the equation of ${{Y^{\prime }}_{A}}^{X_{B}}\;mod\;q$.

Now, $EN_{A}$ can encrypt its datagrams using the new session key which results in $E_{{K^{\prime }}_{A}}$[Audio Data]. Therefore, $EN_{A}$ sends the encrypted datagrams to $EN_{B}$. When $EN_{B}$ receives the encrypted datagrams from $EN_{A}$, it can decrypt those datagrams using the new session key ${K^{\prime }}_{B}$ which results in $D_{{K^{\prime }}_{B}}$[$E_{{K^{\prime }}_{A}}$[Audio Data]] = [Audio Data].

Because $EN_{A}$ changes its secret value, it needs to update its security-related information in the blockchain. $EN_{A}$ updates its state information including the security-related information by sending ToALL Tx to the SN.

## 4. Improvement Effects of Blockchain-Based Approaches

### 4.1. Comparisons between the Existing Vertical Model and the Proposed BINFM Model for the Pre-Call Mobility and Handover Management

**Figure 11:**
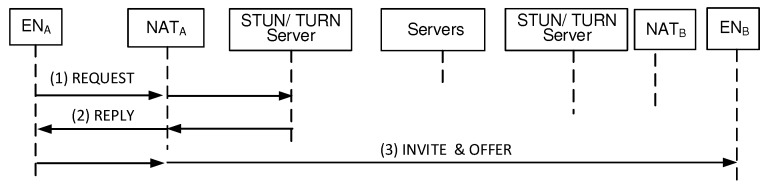
Mid-call mobility management procedure in the vertical model.

Figure 1 shows a series of steps, which correspond to the pre-call mobility management procedure in the vertical model, to complete a secure session set up between two ENs where they are located within the different private networks. Here, each EN changes its location dynamically. Figure 11 shows a series of steps to handle a mid-call mobility management between two ENs at the circumstance that one of them changes its private IP address.

Figure 8 shows the proposed BINFM-based VoIP call setup procedure which corresponds to the pre-call mobility procedure in the proposed BINFM model. As shown in Figure 9, the mid-call mobility management in the proposed BINFM model is already described.

The following assumptions have been made to perform the comparative analysis with respect to total latency to complete the IM management. Three types of delay components exist, that is,

- $T_{I}$: intra-domain delay caused in intra-domain links,
- $T_{II}$: end-to-end delay caused in end-to-end path,
- $T_{III}$: delay caused to collaborate with the distributed servers, which are spread in inter-domain regions,

where $T_{II}$ = 5$T_{I}$ and $T_{III}$ = 10$T_{I}$. This assumption is based on the blockchain network architecture where the unit delay of $T_{I}$ corresponds to the packet delay to travel from a certain EN to its nearest SN and the end-to-end path between two peers is longer by 5 times compared to the unit delay. Also, $T_{III}$ is assumed to be twice compared with the end-to-end path delay because the delay of $T_{III}$ includes delay components needed for searching processes in the distributed servers. Considering that with 4G networks, average latency is around 50 ms, the unit delay of $T_{I}$ is set to 40 ms.

Table 1 compares the vertical model in Figure 1 with BINFM model in Figure 8. In the BINFM system, the Query/Reply procedures are only required to agree on necessary parameters to solve the issues relating to NAT, mobility and security management. As shown in Table 1, the pre-call mobility management latency requires 760 ms in the BINFM system compared to the vertical model, which needs the latency of 1440 ms for pre-call mobility management.

As shown in Table 2, the vertical model yields a latency of 280 ms for mid-call mobility management. In the BINFM model, a mid-call mobility management needs the latency of 200 ms.

### 4.2. Comparisons between the Existing Vertical Model and the Proposed BINFM Model for the Security Management

As shown in Table 3, the BINFM model needs the latency of 200 ms to complete a new key agreement procedure between two peers during a session. the vertical model yields a latency of at least 400 ms for the same key management.

### 4.3. Comparisons between the Existing Vertical Model and the Proposed BINFM Model for the Signaling Overhead

This subsection analyzes the signaling overhead that is imposed in the overall system. Without loss of generosity, three types of signaling overhead can be assumed,

- $S_{I}$: intra-domain signaling overhead caused in intra-domain links,
- $S_{II}$: end-to-end signaling overhead caused in end-to-end path,
- $S_{III}$: signaling overhead caused to collaborate with the distributed servers, which are spread in inter-domain regions,

where $S_{II}$ = 5$S_{I}$ and $S_{III}$ = 10$S_{I}$. This condition is based on the same assumption to obtain the results shown in Table 1. The unit signaling overhead of $S_{I}$ corresponds to the amount of signaling overhead for the Query message in Figure 8 to complete a mission. Inferring using the same method as Table 1, the overall signaling overhead requires 19$S_{I}$ in the BINFM system compared to the vertical model, which needs the overall signaling overhead of 36$S_{I}$ for completing the whole management to establish a secure session. It is found that the signaling overhead, which is imposed in the BINFM system, can be reduced to the level of 52% compared to the vertical model.

### 4.4. Complexity Analysis of the Proposed BINFM Model

If our BINFM approach can be implemented in real time or close to real time within a realistic networking environment, the complexity of the system can be explained in two ways. In the BINFM system, the role of the blockchain network is to enable two ENs as session initiator and session responder to agree on the mutual transport address-related and security-related information close to real-time. Therefore, blockchain information need to be updated as fast as possible when a certain EN issues a ToALL Tx. This latency is the same as the block creation period to extend a new block in the blockchain. Therefore, the latency to complete the registration process will be reduced as much as the block creation period decreases. In this paper, it is required to solve the complexity of the BINFM system in which the proof-of-work takes 1 second on average to succeed. Next, complexity is related to the Query/Reply mechanism. It starts to work by sending a Query Tx to the nearest SN. Then, the SN searches the corresponding transaction data from its blockchain with the Tx Search Table (TST)’s help and replies the searched transaction information. As the number of ENs increases and their movements increase, the complexity of finding information of the desired counterpart will increase. This complexity is closely related to the scalability of the system. It is beyond the scope of this paper.

## 5. Conclusions

Currently, the vertical model is typically used to solve Network Address Translation (NAT), mobility, and security issues for the mobile IoT devices where IPv4 and IPv6 are used together as a network layer protocol. However, the existing vertical model confronts with limitations in handling NAT, mobility and security management in batch. This paper proposed a Blockchain-based Integrated Management system where the the NAT and mobility management are handled together with the security management at once. This paper proved that our BINFM scheme is advantageous in terms of using the blockchain and Query/Reply mechanism, and each side can easily obtain the necessary parameters of the other side required to handle the NAT, mobility, and security management to establish and maintain a secure session between two peers which entered two different private networks. It was proved that the proposed scheme performs better from the viewpoints of pre-call mobility, mid-call mobility, pre-call security, and mid-call security control issues than the existing vertical model.

## Figures and Tables

**Table 1:** Latency comparison for pre-call mobility management.

	BINFM Model	Vertical Model
	(Figure 8)	(Figure 1)
**Delay**	$T_{I}$: (a), (b), (d), (e)	$T_{I}$: (1), (2), (3), (5), (6), (7)
**Components**	$T_{II}$: (c), (f), (g)	$T_{II}$:(8), (9), (10), (11)
	$T_{III}$: None	$T_{III}$: (4)
**Latency**	$4T_{I}+3T_{II}$	$6T_{I}+4T_{II}+T_{III}$
	(760 ms)	(1440 ms)

**Table 2:** Latency comparison for mid-call mobility management.

	BINFM Model	Vertical Model
	(Figure 9)	(Figure 11)
**Delay**	$T_{I}$: None	$T_{I}$: (1), (2)
**Components**	$T_{II}$: (1)	$T_{II}$: (3)
**Latency**	$T_{II}$	$2T_{I}+T_{II}$
	(200 ms)	(280 ms)

**Table 3:** Latency comparison for security management.

	BINFM Model	Vertical Model
	(Figure 10)	(Figure 1)
**Delay Components**	$T_{II}$: (a)	$T_{II}$: (10), (11)
**Latency**	$T_{II}$	$2T_{II}$
	(200 ms)	(400 ms)
